# Social Context Considerations for Future HIV Vaccine Introduction and Implementation

**DOI:** 10.3390/vaccines14050450

**Published:** 2026-05-19

**Authors:** Nivedita L. Bhushan, Rafael Gonzalez, Brian G. Southwell

**Affiliations:** 1 RTI International, Durham, NC 27709, USA; 2 Fred Hutchinson Cancer Center, Seattle, WA 98109, USA

**Keywords:** HIV vaccine, mental model, social context, communication, behavior

## Abstract

**Background:** The development of an efficacious preventive human immunodeficiency virus (HIV) vaccine remains a central goal of global HIV elimination efforts, yet biological performance alone will not determine a future vaccine’s public health impact. **Method:** This review draws on behavioral science, communication research, vaccine implementation, and HIV prevention literature to identify cognitive, social, and structural challenges that are likely to shape public acceptance and uptake of a future HIV vaccine, as well as to outline evidence-based opportunities for addressing them. **Results:** Based on the available literature, mental models of both HIV and vaccination will be a critical determinant of how communities consider a future vaccine, particularly given that emerging mRNA and adjuvanted platforms may generate side effects that could be easily misinterpreted and that highly effective long-acting pre-exposure prophylaxis (PrEP) options already exist and will shape how individuals evaluate a vaccine’s relative value. HIV-related stigma further complicates this landscape by making vaccination a socially interpreted behavior, unlike some other vaccination efforts. Together, these factors suggest that hesitancy and misalignment between public understanding and scientific evidence are predictable and should be anticipated rather than addressed reactively. At the same time, decades of HIV prevention implementation research have established an evidence base for vaccine communication, and existing community engagement infrastructure offers a foundation upon which future rollout efforts can build. We highlight three evidence-based strategies as particularly promising levers for encouraging acceptance and adoption. **Conclusions:** We conclude with recommendations for HIV vaccine researchers and healthcare professionals to invest in formative research, build community partnerships in advance of vaccine availability, and pilot integrated delivery models within existing HIV prevention services.

## 1. Introduction

The prospect of preventive HIV vaccines offers tremendous potential benefit for society while also posing complex challenges involving the social diffusion of new medical technologies, existing patterns of information access, and human cognitive and behavioral tendencies. To inform planning for future implementation of the distribution of what are currently vaccines in development, we can outline what we know about the ways in which existing societal patterns may constrain or facilitate future vaccine acceptance. To inform planning for future implementation of HIV vaccines currently in development, we outline what is known about the ways in which existing societal patterns may constrain or facilitate future vaccine acceptance. We do this through a narrative review of current directions in vaccine research and relevant social science literature and exploration of social and behavioral factors likely to shape community acceptance of future HIV vaccine technologies.

The development of pre-exposure prophylaxis (PrEP) has fundamentally transformed global HIV prevention efforts [1]. Since its United States Food and Drug Administration (FDA) approval in 2012, PrEP has evolved in its duration of therapeutic action, modality options, and effectiveness [2,3]. The most recent PrEP product is twice-yearly, injectable lenacapavir, which has demonstrated high efficacy in clinical trials across diverse populations [3,4,5,6]. Despite this progress, new HIV infections persist and a preventive HIV vaccine is still widely regarded as essential for ending the HIV epidemic [7]. Several large-scale trials (e.g., HVTN 702, HVTN 705, HVTN 706, RV144, PrEPVacc, AMP (HVTN 703/704, HPTN 081/085)) have evaluated a range of vaccine strategies, but none have yet demonstrated sufficient or durable efficacy for widespread implementation [8]. When an efficacious HIV vaccine does emerge, its public health impact will depend on its biological performance as well as the scientific community’s ability to demonstrate and communicate its value. Unlike other instances of vaccine declination or hesitancy discussed in existing literature, future HIV vaccine acceptance may be uniquely shaped by the intersection of HIV-related stigma, emerging vaccine technologies such as mRNA platforms, and comparison to already available biomedical tools such as PrEP [9,10,11]. This challenge will be complicated by two current realities: (1) well-tolerated, highly efficacious PrEP already exists and (2) next generation mRNA and adjuvanted vaccines may cause transient reactogenicity. Although such side effects would largely be indicative of immune activation, they could substantially impact HIV vaccine acceptance and adoption, behavior which is influenced by public understanding of HIV processes and vaccines.

Public understanding comprises not simply information available to people but also existing mental models and the cognitive frameworks individuals use to process information, evaluate risk, and guide decision-making [12,13,14]. Prior research in health communication and risk perception suggests that when new information is inconsistent with existing mental models, individuals are more likely to rely on heuristics, affective responses, or social cues rather than deep analytic processing [15,16,17]. Dual process theory specifically posits that individuals often default to fast, heuristic-driven processing when they are facing uncertainty, stigma, or information overload, which are well-documented features of the public’s response to emerging infectious diseases and novel vaccines [16,18,19,20,21,22,23]. For example, public understanding of the Zika virus in Guatemala was anchored to previous experiences with endemic mosquito-borne diseases like dengue and chikungunya, causing individuals to overlook the virus’s novel sexual transmission pathway [18]. Similarly, early in the COVID-19 pandemic, individuals’ willingness to obtain a novel coronavirus vaccine was largely influenced by their past influenza vaccination behaviors [18]. These findings suggest that the public will interpret a future HIV vaccine through pre-existing frameworks shaped by past exposures and experiences, as well as an ever-evolving, saturated information environment.

The contemporary information environment likely will introduce complexity to the already challenging task of rolling out a future preventive HIV vaccine. High volumes of health content, algorithmic reinforcement of popular opinion, and misinformation can amplify heuristic processing and widen the gap between public mental models and scientific evidence [22,24,25,26,27]. A future HIV vaccine will face an information environment shaped by stigmatized misconceptions of HIV transmission and risk, narratives associating new vaccines with past vaccine controversies, and conflicting messaging on new versus established PrEP modalities. Furthermore, prior research suggests that when individuals are unfamiliar with a new biomedical tool’s characteristics (e.g., how it works, expected side effects, novel modalities, advantage over existing options), the information gap is a significant barrier to acceptance and adoption [9,10,28,29,30]. Addressing these challenges will require strategies that account for pre-existing frameworks and information gaps to ensure that future HIV vaccine efforts are successful. Importantly, successful implementation may look different across settings depending on regional epidemiology, healthcare infrastructure, and the extent to which HIV vaccination can be integrated into existing prevention and immunization systems [31,32,33,34,35,36].

The present commentary reviews available behavioral science, vaccine implementation, and HIV prevention literature to identify the challenges and opportunities that public health professionals, healthcare providers, and policymakers will face in planning for HIV vaccine rollout once an efficacious product becomes available. Consideration of how people think and behave as well as consideration of what we know about vaccine science, public health, and healthcare implementation is relevant to planning for future HIV vaccine promotion.

## 2. Challenges

The COVID-19 pandemic demonstrated that vaccine effectiveness alone does not guarantee vaccine acceptance or uptake and that vaccine hesitancy is shaped by a multilevel array of factors (e.g., structural barriers to access, distrust of healthcare systems, exposure to misinformation, concerns about safety and side effects, misconceptions about efficacy, perceived disease severity) [28,37,38]. Some drivers of vaccine hesitancy (or the extent to which a population remains unvaccinated) can be addressed through clear, sustained messaging while others will require structural solutions. Behavioral science similarly cautions that messaging alone is unlikely to overcome barriers that are fundamentally structural or experiential in nature, and that overreliance on messaging to address such challenges may erode trust if expectations diverge from lived experience. Distinguishing between these types of challenges is therefore critical for understanding the limits of behavioral interventions and for setting realistic expectations for future HIV vaccine implementation. Thus, we have organized challenges into four domains: cognitive and interpretive, social and relational, product-specific, and structural.

### 2.1. Cognitive and Interpretive Challenges

Public understanding of HIV vaccines will be shaped by existing mental models of both vaccination and HIV, which may not align with current and emerging models of vaccine function. Vaccines are often conceptualized as one-time or infrequent interventions typically administered during childhood or seasonal campaigns, and associated with mild side effects and minimal social stigma [28]. In contrast, HIV is widely understood as a chronic and stigmatized condition requiring ongoing treatment and medical monitoring [11,23]. A future HIV vaccine, particularly one that requires multiple doses or involves moderate side effects, may disrupt both of these mental frameworks. Prior research demonstrates that individuals often rely on outdated models of vaccine function, including beliefs that HIV vaccines may contain live virus [39]. These mismatches between scientific mechanisms and public understanding can lead to uncertainty. This challenge may be amplified in the context of emerging platforms like mRNA or vector-based technologies, in which general population understanding of effect mechanisms is understandably limited. While existing research has documented general vaccine misconceptions, researchers have paid less attention to how novel vaccine platforms and necessary dosing strategies may contrast with existing mental models in the context of HIV prevention [10,28]. Together, these gaps between scientific reality and public mental models pose a meaningful barrier to acceptance and uptake that will need to be proactively addressed.

### 2.2. Social and Relational Challenges

HIV vaccination is likely to operate as a socially interpreted behavior that is embedded within existing stigma structures and identity frameworks. Stigma literature suggests that individuals often evaluate medical decisions not only through personal risk assessment but through anticipated social judgment, particularly when the health condition carries moral or identity-based associations [9,11,40,41]. In contrast to routine vaccines that are often framed as universally relevant or appropriate at certain developmental stages, HIV vaccination is more likely to be interpreted through a social lens that assigns meaning to who is vaccinated and why they are getting vaccinated. Because HIV stigma remains persistent across many communities, HIV vaccination may also serve as an unwanted disclosure, signaling “high-risk” sexual behavior or affiliation with marginalized groups. This dynamic distinguishes HIV vaccination from nearly all other vaccine-preventable conditions, where the social meaning of vaccination is either neutral or positive. This positionality, where the act of vaccination itself carries social meaning, will require proactive mitigation strategies when HIV vaccines are rolled out.

### 2.3. Product Challenges

Side effect concerns represent a significant challenge for future HIV vaccine uptake, particularly for mRNA and adjuvanted models whose reactogenicity profiles may amplify existing public anxieties. Risk perception research demonstrates that rare events associated with severe or poorly understood outcomes tend to elicit responses different from those triggered by common, transient side effects like fatigue or injection site pain and that such perceptions are resistant to purely statistical reassurance [31]. The myocarditis cases reported during COVID-19 mRNA vaccination illustrate this dynamic. Despite low incidence and generally favorable outcomes, the condition’s cardiac associations and media visibility established a collective reference point likely to shape how publics interpret side effect risk for future vaccines [42,43,44]. Thus, side effects must be understood not only as biomedical phenomena but as socially interpreted signals that shape trust and decision-making [25]. Future HIV vaccine messaging must be transparent and strike balance between benefit and cost without either minimizing or inadvertently reinforcing legitimate side effect concerns.

The introduction of highly effective pre-exposure prophylaxis (PrEP) options, including long-acting injectable formulations such as lenacapavir, fundamentally alters the perceived value proposition of a future HIV vaccine [4,5,29,30]. Unlike earlier prevention landscapes, individuals may evaluate HIV vaccination alongside existing biomedical tools that already offer high levels of protection [28]. As a result, decisions about vaccine uptake are likely to be shaped not only by vaccine efficacy, but by how individuals perceive its relative advantage, convenience, and fit within their daily lives. While long-acting PrEP reduces adherence burden compared to daily oral regimens, it still requires ongoing engagement with healthcare systems, periodic injections, and a continued identification of oneself as being at risk of HIV exposure [9]. These requirements may be challenging in contexts where risk perception fluctuates over time, healthcare access is inconsistent, or stigma associated with HIV prevention remains high [9,11]. In contrast, vaccines are often perceived as more definitive or time-limited interventions, which may appeal to individuals seeking protection without sustained behavioral or clinical engagement.

At the same time, PrEP and HIV vaccines may not be experienced as interchangeable options. PrEP delivery has historically focused on specific populations identified as higher risk, which can reinforce stigma and create gaps in access for individuals who do not perceive themselves to fit those categories [45]. In contrast, vaccination programs have the potential to operate at a broader population level, leveraging existing immunization infrastructure and potentially reaching individuals who are not currently engaged in HIV prevention services [28]. These differences suggest that the role of a future HIV vaccine will be shaped not only by its biological performance, but by how it is positioned within an existing prevention ecosystem. Importantly, many of the factors influencing this positioning are structural in nature and may not be fully addressable through communication strategies alone [32,33,46,47].

### 2.4. Structural Challenges

Structural factors (e.g., healthcare access, delivery systems, cost, and eligibility criteria) will shape not only whether a vaccine reaches priority populations, but how those populations perceive and respond to it. In low- and middle-income countries (LMICs), where the HIV burden is often highest, health systems may struggle to accommodate the cold-chain logistics, manufacturing scale-up demands, multidose regimen requirements, and healthcare workforce capacity needed for novel vaccine delivery [34,35,36]. At the macroeconomic level, policymakers will need to navigate complex financing and procurement gaps, including the eligibility constraints of existing global health funding vehicles (such as vaccine alliance known as Gavi), to ensure that HIV vaccine introduction complements, rather than competes with or displaces, existing funding streams for PrEP and antiretroviral therapies [32,33]. Regulatory capacity in high-burden countries represents an additional structural barrier. WHO prequalification pathways and coordinated multi-stakeholder regulatory review processes will be essential to ensure that an efficacious vaccine can be approved and deployed expeditiously across the settings that need it most [32,33,48]. Finally, at the administrative level, tracking vaccine uptake introduces critical privacy concerns; maintaining secure vaccination records will be essential to protect patient confidentiality, as record of receiving an HIV vaccine may carry risks of both inadvertent HIV status disclosure and unwanted association with stigmatized populations or behaviors [38,49,50]. Access and eligibility concerns, including confusion about who qualifies to receive the vaccine and whether HIV-positive individuals are eligible, are likely to further complicate uptake in populations that stand to benefit most [38,51,52].

Importantly, structural challenges affecting HIV vaccination are unlikely to operate uniformly across settings globally. HIV epidemics vary considerably in their epidemiology, healthcare infrastructure, financing mechanisms, and sociopolitical context [32,33,34,35,36]. In settings with generalized epidemics and established HIV service infrastructure, integration of HIV vaccination into existing prevention and treatment systems may be more feasible, whereas regions with concentrated epidemics, fragmented healthcare access, or heightened stigma toward marginalized populations may face distinct implementation barriers [33,34,35,36]. These differences suggest that future HIV vaccine rollout strategies will require regional adaptation rather than a singular global implementation approach.

## 3. Opportunities

The behavioral science, vaccine implementation, and HIV prevention literature also offer a range of evidence-based strategies to support future HIV vaccine acceptance and uptake. These opportunities are most powerful when understood not as isolated tactics but as complementary approaches that, taken together, can address the cognitive, social, structural, and product barriers previously discussed while also acknowledging individual perspectives and community needs.

### 3.1. Community Engagement

Community engagement represents one of the most essential strategies for ensuring global acceptance and adoption of novel HIV prevention tools, particularly amongst populations who have been historically underserved by health systems and underrepresented in biomedical research [53,54,55]. Given that public mental models of both HIV and vaccines are shaped by lived experience, trust, and community context, effective vaccine rollout will require strategies that engage communities as active participants in public health messaging [11,41,56]. Notable examples of community engagement strategies in the vaccine context include culturally concordant, multilingual vaccination clinics that reduce access barriers for marginalized groups, as well as participatory design approaches that leverage iterative collaboration to co-create uptake strategies, as successfully demonstrated by COVID-19 efforts with Latino youth [57,58,59]. The HIV clinical trials literature also offers numerous evidence-based engagement strategies developed through decades of navigating community mistrust and stigma. Effective engagement practices in HIV vaccine trials have included community education through external stakeholder partnerships, the use of community advisory boards as ongoing bridges between researchers and communities, co-development of recruitment messages, communication strategies that prioritize plain language, community leader endorsement, and dissemination through both traditional and social media [39,60,61,62]. Taken together, these evidence-based community engagement strategies underscore that sustained, ongoing partnerships, rather than transient outreach, will be essential to overcoming pervasive stigma and mistrust, addressing side effect concerns, and communicating the added value of a future HIV vaccine alongside highly effective PrEP.

### 3.2. Bundled Interventions

Bundled interventions offer another important opportunity for future HIV vaccine rollout, particularly given that the challenges identified in our review (e.g., understanding of vaccine processes, stigma, side effect concerns, availability of highly effective PrEP) are unlikely to be addressed by a singular strategy and each represents a dimension along which public mental models may misalign with a future HIV vaccine [28,37]. Public health implementors have historically included both clinically focused and patient focused strategies within bundled interventions. Clinically focused bundles embed evidence-based strategies within existing healthcare workflows to reduce missed vaccination opportunities [28,63,64]. For example, human papillomavirus (HPV) vaccination increased amongst young women when clinics integrated staff champions, offered no-cost vaccines, eliminated pre-vaccination pregnancy tests, pre-screened patient charts, used nurse-led recommendations, and incorporated clinical note prompts [65]. Bundling vaccines with both preventive and curative services also has proven effective—flu vaccines are routinely offered during chronic disease and prenatal visits, and hepatitis B vaccines are frequently administered during clinical encounters related to sexually transmitted infection (STI) or substance use [66,67,68]. Integrating HIV vaccine delivery into similar clinic workflows has the potential to correct mental model misalignment by using patient–clinic engagement to address misconceptions about side effects, new modalities, and the comparative value of an HIV vaccine at the point of care. For example, co-administering an HIV vaccine within existing HIV prevention services (e.g., PrEP initiation visits, STI screening, sexual health consultations) could normalize HIV vaccination within established care pathways and reduce the stigma associated with seeking it independently [45]. Patient-focused bundles complement clinical strategies by targeting the lived experience of vaccination through tailored educational outreach, appointment reminders, logistical support, and decision-making aids that build confidence and autonomy [28,64,69,70,71,72]. Together, bundled intervention planning that addresses both system-level delivery and individual-level decision-making will be essential to achieving equitable and widespread future HIV vaccine uptake.

### 3.3. Messaging Strategies

Development of appropriate messaging strategies represent a third critical opportunity for future HIV vaccine rollout and yet success in message framing is not guaranteed and potential missteps loom large. The framing and structure of vaccine messages have been shown to have a meaningful impact on vaccine acceptance because messages can intentionally address misalignment between mental models and the information environment [72,73,74,75]. One factor to consider in messaging strategy is message sidedness, the degree to which communication acknowledges opposing viewpoints. While one-sided messages present only supporting arguments, two-sided refutational messages directly acknowledge and then counter opposing beliefs, an approach that has demonstrated effectiveness and potential destigmatizing effects among individuals who are neutral or opposed to a health intervention [76,77,78,79]. Given the well-documented stigma surrounding both HIV and vaccination, two-sided refutational messaging may be especially valuable in HIV vaccine messaging by validating concerns about potential side effects, novel modalities, or perceived redundancy with PrEP options. By systematically addressing these concerns, there is the potential to counter the heuristic-driven resistance that misaligned mental models can produce. Another strategic approach to consider is pre-bunking which involves proactively addressing anticipated misconceptions and barriers before audiences encounter such claims and they become entrenched [80]. This approach has demonstrated effectiveness in contraceptive and influenza vaccine contexts and is particularly well-suited to HIV vaccine rollout, where side effect and stigma-related concerns are predictable and can be anticipated and addressed in advance of widespread vaccine availability [81,82,83]. A third effective messaging strategy is tailoring gain-framed or loss-framed messages for specific audiences and their established risk perceptions [84]. To introduce a future HIV vaccine, tailoring frame selection to existing mental models will be essential. Individuals who perceive themselves as low-risk or existing PrEP users may respond more readily to gain-framed messages emphasizing community-level protection, while those whose mental models are shaped by HIV stigma or negative healthcare experiences may be more responsive to loss-framed messages emphasizing the personal consequences of remaining unvaccinated [9,84,85,86,87]. Because people tend to share information about stigmatized health conditions less than other conditions [88], framing discussion about HIV as being widely relevant and socially acceptable also may encourage greater willingness among some people to share information about HIV vaccines with their social networks than otherwise might be the case. Lastly, acknowledging uncertainty associated with biomedical and public health research and explaining the scientific process used to test hypotheses can encourage perceived trustworthiness of scientific institutions, meaning messages that offer transparency regarding the process of vaccine development may bolster message credibility [89]. Together, these evidence-based messaging strategies underscore that proactively encouraging mental model alignment and anticipating information environment challenges, rather than reacting to misinformation and perceived hesitancy after the introduction of new vaccines, will be essential in efforts to achieve HIV vaccine acceptance and adoption.

## 4. Implications

The challenges and opportunities outlined in this review collectively suggest that the behavioral and social dimensions of HIV vaccine rollout deserve as much deliberate planning as the biological ones. The success of a future HIV vaccine will depend not only on its efficacy but on the extent to which its characteristics align with existing public mental models, social contexts, and structural realities of healthcare delivery. Although social science offers a range of strategies to support vaccine acceptance, these approaches must be applied with an understanding of their limits. Some barriers, particularly those rooted in structural conditions or product characteristics such as dosing schedules or side-effect profiles, may not be fully addressable through communication alone.

## 5. Limitations

This manuscript is a narrative, conceptually oriented review of relevant literature. Given that we did not formally employ systematic review methods, we must acknowledge the potential for selection bias in the literature considered. Our objective was not to comprehensively catalog all existing studies, but rather to draw together insights from behavioral science, communication research, vaccine implementation, and HIV prevention to identify challenges and opportunities relevant to future HIV vaccine rollout. As empirical data on HIV vaccine implementation remain limited in the absence of an efficacious product, our conclusions offer considerations for HIV vaccine rollout rather than to provide policy guidance based on comprehensive data regarding HIV vaccination uptake. Future work should explore hypotheses and questions suggested here.

## 6. Conclusions and Future Directions

Translating an efficacious HIV vaccine into meaningful public health impact will require coordinated action across research, health systems, communication, and community engagement. First, HIV researchers and public health practitioners should proactively integrate behavioral social science considerations into vaccine development and rollout planning, rather than treating them as downstream communication challenges. Anticipating how individuals will interpret novel vaccine modalities, side effects, and comparative value relative to existing prevention tools such as PrEP will be essential for shaping effective uptake strategies. Second, healthcare systems and policymakers should prioritize delivery models that reduce the social and logistical burden of vaccination, including integration into existing clinical workflows and HIV prevention services. Bundled intervention approaches that combine clinical, structural, and patient-centered strategies may be particularly effective in addressing the multilevel barriers identified in this review. Third, communication strategies should move beyond information provision to actively address misalignment between scientific evidence and public mental models. Approaches such as two-sided messaging, pre-bunking, tailored framing, and acknowledgment of the scientific process offer opportunities to engage with anticipated concerns about side effects, stigma and perceived redundancy with PrEP in a way that builds trust rather than eroding it. Finally, sustained community engagement should be treated as foundational rather than supplemental to HIV vaccine implementations efforts. Building trust, co-developing messaging, and leveraging community partnerships will be critical for addressing stigma, increasing accessibility, and ensuring that vaccination efforts are responsive to diverse lived experiences. For scientific progress to translate into meaningful public health impact, we must lay the groundwork before an efficacious vaccine arrives.

## Data Availability

The authors generated no new data in conducting this review.

## Abbreviations

The following abbreviations are used in this manuscript:

FDA	Food and Drug Administration
HIV	human immunodeficiency virus
HPV	human papillomavirus
PrEP	pre-exposure prophylaxis
